# Construction of ddRADseq-Based High-Density Genetic Map and Identification of Quantitative Trait Loci for Trans-resveratrol Content in Peanut Seeds

**DOI:** 10.3389/fpls.2021.644402

**Published:** 2021-03-18

**Authors:** Huaiyong Luo, Jianbin Guo, Bolun Yu, Weigang Chen, Huan Zhang, Xiaojing Zhou, Yuning Chen, Li Huang, Nian Liu, Xiaoping Ren, Liying Yan, Dongxin Huai, Yong Lei, Boshou Liao, Huifang Jiang

**Affiliations:** Key Laboratory of Biology and Genetic Improvement of Oil Crops, Ministry of Agriculture, Oil Crops Research Institute of the Chinese Academy of Agricultural Sciences, Wuhan, China

**Keywords:** peanut, resveratrol, high-density genetic map, QTLs, single nucleotide polymorphisms

## Abstract

Resveratrol (trans-3,4′,5-trihydroxystilbene) is a natural stilbene phytoalexin which is also found to be good for human health. Cultivated peanut (*Arachis hypogaea* L.), a worldwide important legume crop, is one of the few sources of human's dietary intake of resveratrol. Although the variations of resveratrol contents among peanut varieties were observed, the variations across environments and its underlying genetic basis were poorly investigated. In this study, the resveratrol content in seeds of a recombination inbred line (RIL) population (Zhonghua 6 × Xuhua 13, 186 progenies) were quantified by high performance liquid chromatography (HPLC) method across four environments. Genotypes, environments and genotype × environment interactions significantly influenced the resveratrol contents in the RIL population. A total of 8,114 high-quality single nucleotide polymorphisms (SNPs) were identified based on double-digest restriction-site-associated DNA sequencing (ddRADseq) reads. These SNPs were clustered into bins using a reference-based method, which facilitated the construction of high-density genetic map (2,183 loci with a total length of 2,063.55 cM) and the discovery of several chromosome translocations. Through composite interval mapping (CIM), nine additive quantitative trait loci (QTL) for resveratrol contents were identified on chromosomes A01, A07, A08, B04, B05, B06, B07, and B10 with 5.07–8.19% phenotypic variations explained (PVE). Putative genes within their confidential intervals might play roles in diverse primary and secondary metabolic processes. These results laid a foundation for the further genetic dissection of resveratrol content as well as the breeding and production of high-resveratrol peanuts.

## Introduction

Resveratrol is a non-flavonoid polyphenol compound synthesized and functioned as phytoalexinin more than 70 plant species (Pastor et al., 2019). Natural resveratrol exists mainly in the trans-isoform (Novelle et al., 2015) and has been identified in only a few of edible plants such as grape, peanut, and berries (Pastor et al., 2019; Singh et al., 2019). Resveratrol is synthesized by stilbene synthase (STS) *via* the phenylalanine/polymalonate biosynthetic pathway (Shomura et al., 2005). The dietary intake of resveratrol from moderate red wine was reported to be partly contributed to the “French paradox” in the early 1990s, and studies have been conducted to increase the contents of resveratrol in edible plants (e.g., grapes) which could be produced to health foods (Hasan and Bae, 2017). In addition, the favorable effects of resveratrol for human health have been reported in the therapeutic outcomes in many preclinical and clinical trials of cardiovascular diseases, cancer and other important human diseases, although inconsistent results were observed in other studies (Fogacci et al., 2019; Singh et al., 2019; Wang et al., 2019b).

As one of the dietary sources of resveratrol (Delaunois et al., 2009), the cultivated peanut (*Arachis hypogaea* L.) is an important legume crop grown in more than 100 countries (FAOSTAT, 2019). Its global productions were around 47 million tones in recent years (FAOSTAT, 2019), and its seeds are consumed worldwide in diverse forms, such as nuts, peanut butter, edible oil, and candies (Varshney et al., 2013). The resveratrol in peanut mainly exists in the trans-form, and the HPLC method has been developed to quantify its content. Significant variations were observed for resveratrol contents among different peanut varieties (Sanders et al., 2000). Using the US mini-core collection as materials, Wang et al. (2013) found that there was a nine-fold difference (30–260 μg/kg) of resveratrol content in seeds across different peanut accessions. However, this study was conducted in single environment, and the variation of resveratrol content across environments was not evaluated. In addition, the genetic basis for these variations was poorly investigated in peanut as well as other plant species producing resveratrol. Therefore, more efforts should be made in genetic studies to provide the theoretical basis and technique support for breeding and production of novel high resveratrol varieties.

QTL mapping using bi-parental genetic population has become a routine approach to dissect the genetic basis for quantitative traits, such as yield-related traits, resistance to diseases, tolerance to drought, oil content and fatty acid compositions (Varshney et al., 2009; Vishwakarma et al., 2017). However, the construction of high-quality and high-density genetic map is the prerequisite for the discovery of genomic-wide QTL. With the reducing of high-throughput sequencing cost, the SNP polymorphisms have been utilized as markers to construct high-density genetic maps (Pandey et al., 2016). Through sequencing based genotyping methods such as ddRADseq (Zhou et al., 2014), GBS (Dodia et al., 2019) and SLAF-seq (Hu et al., 2018), SNP-based genetic maps could be constructed in much shorter time than traditional gel-based genotyping and have lower cost than whole-genome resequencing (Agarwal et al., 2018). However, the previously reported high-density genetic maps were developed though *de-novo* method in peanut (Zhou et al., 2014; Hu et al., 2018; Dodia et al., 2019). Along with the availability of peanut genome sequences (Bertioli et al., 2019; Chen et al., 2019a; Zhuang et al., 2019), it is feasible to construct the high-density genetic map through the reference-based method approach which might benefit in making the best use of sequencing reads and discovering chromosome variations.

In the present study, the resveratrol content in peanut seeds of a RIL population were quantified across four environments. The effects of genotype, environments and genotype × environment interactions on the variation of resveratrol contents in the RIL population were analyzed. Using the recent published peanut genome sequence as reference, a high-density and high-quality genetic map was constructed through the reference-based analysis, upon which nine additive QTL were identified to be associated with resveratrol content in peanut seeds. Functions of the putative genes located in the confidential intervals of the identified QTL were investigated.

## Materials and Methods

### Plant Materials

The RIL population derived from the cross between Xuhua 13 and Zhonghua 6 (Luo et al., 2018) was used as plant materials in the present study. Recently, ddRADseq reads were generated for 186 RILs in F_6_ generation of the RIL population (Liu et al., 2020). Four generations (F_6_ to F_9_) of the RIL population were used for phenotyping resveratrol contents, and they were planted using randomized complete block design with three replications in Wuchang in 2015 (F_6_ generation) and 2016 (F_7_ generation), Yangluo in 2017 (F_8_ generation), and Xiangyang in 2018 (F_9_ generation). These trials were designated as four environments: WC2015, WC2016, YL2017, and XY2018, respectively. Matured pods were harvested, air-dried, stored at 4°C until shelled by hand before phenotyping.

### Phenotyping of Resveratrol Contents by HPLC

The standard *trans-*resveratrol (CAS 501-36-0) was purchased from Sigma (St Louis, MO, USA) and used to generate the standard curve. The resveratrol contents in seeds of the RIL population were quantified according to the reported HPLC method (Wang et al., 2013). Briefly, 10 g of peanut seeds were ground by a coffee blender. Then, 5 ± 0.0001 g fine powder, which passed through a 20 mesh sieve, was used for extraction of resveratrol in a 50-ml centrifuge tube. The tube, into which 40 ml ethanol (85%) was added, was placed in 80°C water bath for 1 h and then centrifuged at 10,000 rpm for 10 min. Subsequently, 8 ml supernatant was filtered through a C18/AL-N SPE column (Agela Technologies). The filtrate was blow-dried with nitrogen gas and dissolved with 1 ml methanol. The sample was finally filtered with a 0.22 μm filter and quantified using the Agilent 1290 system with the C18 column (4.6 mm × 150 mm, 5 mm; Agilent Technologies, USA). The column temperature was set as 30°C. The mobile phase was consisted of (A) ultrapure water with 0.05% acetic acid and (B) HPLC-grade acetonitrile. The chromatogram was recorded at 306 nm and the flow rate was 0.9 ml/min. After 10 μl samples were injected, a 12 min elution was performed with 78% A and 22% B. After that, the column wash was washed for 2 min with 60% A and 40% B to get ready for next injection. The phenotypic distribution of resveratrol contents in the RIL population was plotted by the “ggplot2” R package.

### SNP Calling and Genotyping

The ddRADseq reads of the RIL population (BioProject: PRJNA520741) (Liu et al., 2020) were re-analyzed following the pipeline of reference-based analysis (Rochette and Catchen, 2017) of the Stacks software (Catchen et al., 2013) to identify SNPs. Briefly, low-quality reads were removed by the process_radtags unit of Stacks. Then, high-quality reads from each RIL were mapped to the recently published genome sequences (the KYV3 version) of cultivated peanut cv. Tifrunner (Bertioli et al., 2019) by the BWA software (Li and Durbin, 2009). The gstacks unit of Stacks was used to build loci from uniquely mapped reads. The populations unit was used to remove loci shared by <80% of samples and output site level SNP calls in VCF format. These SNPs were transformed into genotypes using an in-house Perl script. Individual SNPs were recorded as “A” representing homologous for Zhonghua 6, “B” representing homologous for Xuhua 13, and “X” representing heterozygous or missing alleles). In order to conveniently retrieve physical positions, SNPs was designated with initial letters “TIF” (representing Tifrunner), followed by the corresponding chromosome and position. For example, TIF.01:7840610 was the SNP identified at position 7,840,610 bp on chromosome 1 (A01) of the Tifrunner reference genome.

### Construction of High-Density Genetic Map

The identified high-quality SNPs were clustered into bins using a reference-based method, i.e., the SNPs with identical genotypes and alongside each other on the reference genome were clustered as one bin, and the SNP with the least missing genotypes was selected to represent the bin. The genetic map was constructed based on these bins using the QTL IciMapping software (Meng et al., 2015). Pearson's Chi square test was performed to test the goodness of fit to the expected segregation ratio 1:1 for each locus (*P* < 0.01).

### Identification of QTL for Resveratrol Content

Additive QTL were identified by the composite interval mapping (CIM) method of the WinQTLCart software (Wang et al., 2012) with default parameters for each environment. The BLUP values for resveratrol contents across the four environments were calculated using the “lme4” R package and used to identify QTL as well. The identified QTL were designated with initial letters “*q*,” followed by trait name (*RES* for resveratrol content) and the corresponding chromosome. A number were added if two or more QTL were identified on the same chromosome. For example, *qRESB07.1*and *qRESB07.2* were the first and second QTL identified on chromosome B07, respectively. The putative genes within the 2 LOD confidential interval of identified QTL were retrieved from the genomic annotation of the peanut accession Tifrunner (Bertioli et al., 2019). The annotations of the identified genes were obtained from the Tifrunner annotation file. Kyoto Encyclopedia of Genes and Genomes (KEGG) analysis of the putative genes were conducted using the Blast2GO suite (Gotz et al., 2008).

## Results

### Phenotypic Variation of Resveratrol Contents in the RIL Population

**Figure 1:**
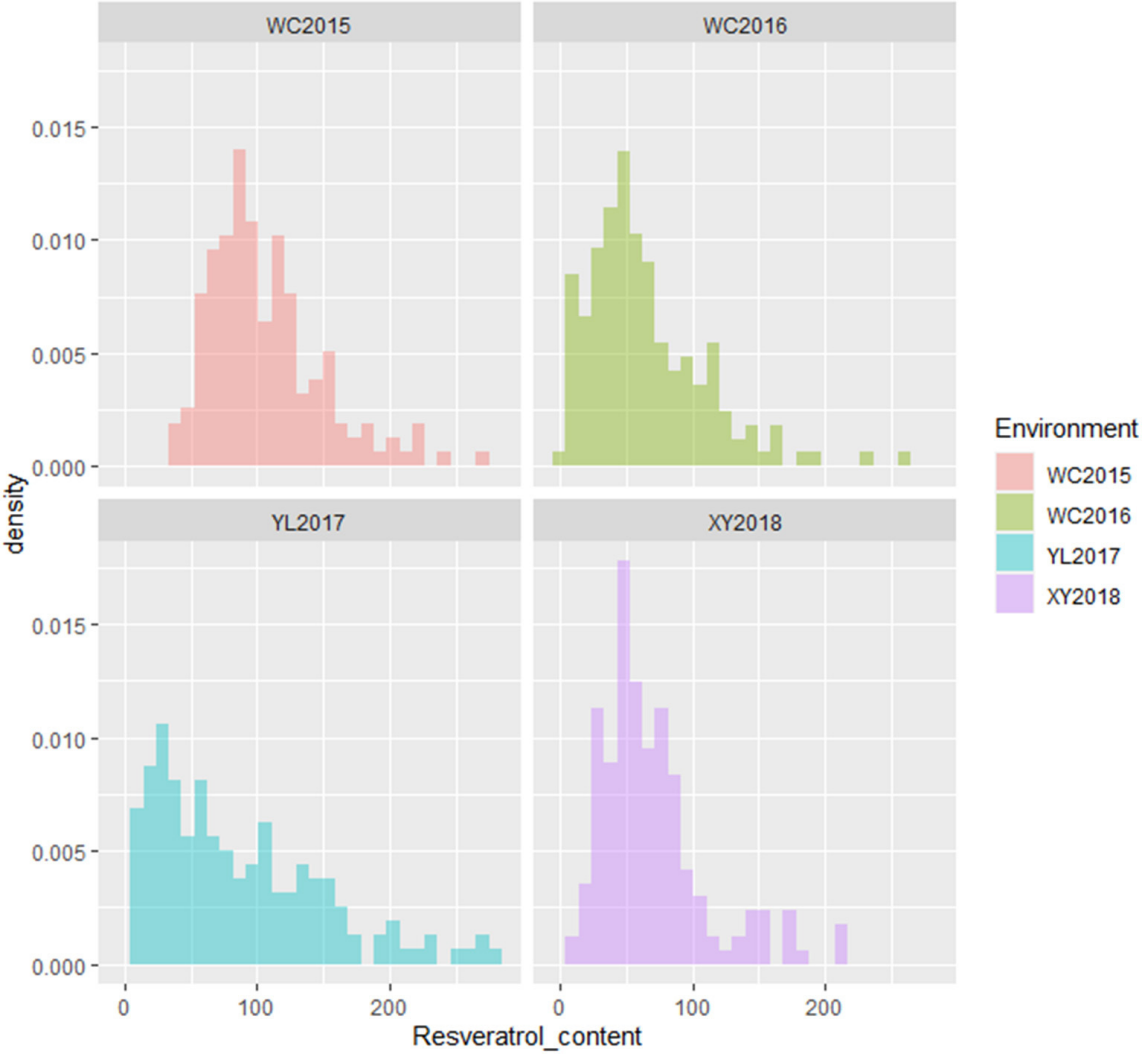
Phenotypic distribution of resveratrol contents in the RIL population across environments. The *y*-axis represented density, while the *x*-axis represented values of resveratrol contents (μg/kg).

The resveratrol content of the variety Xuhua 13 (the female parent) was consistently lower than that of the variety Zhonghua 6 (the male parent) across the four environments including WC2015, WC2016, YL2017 and XY2018 (Figure 1, Table 1). The resveratrol contents of the 186 RILs varied from 37.33 to 270.00 μg/kg in WC2015, from 3.61 to 258.79 μg/kg in WC2016, from 8.96 to 282.89 μg/kg in YL2017, and from 13.60 to 215.06 μg/kg in XY2018 (Table 1). As shown in Figure 1 and Table 1, the distribution of resveratrol contents in the RIL population was continuous and with transgressive segregation. According to the variance analysis across the four environments, genotypes, environments and genotype × environment interactions significantly influenced resveratrol contents among RILs (Supplementary Table 1, *P* < 0.001). The broad sense heritability of resveratrol content in the present study was estimated to be 0.33, indicating that environments had significant influence in resveratrol contents of peanut seeds.

**Table 1:** Descriptive statistics of resveratrol contents in the RIL population across four environments.

**Environment**	**Xuhua 13**	**Zhonghua 6**	**Range**	**Mean**	**SD**	**Skewness**	**Kurtosis**
WC2015	58.77	116	37.33–270.00	106.94	43.03	1.123	1.386
WC2016	40.6	92.36	3.61–258.79	64.54	44.54	1.347	2.601
YL2017	65.59	96.82	8.96–282.89	85.74	63.56	1.009	0.551
XY2018	44	91.18	13.60–215.06	69.08	39.09	1.43	2.169

### Genotyping of the RIL Population

**Figure 2:**
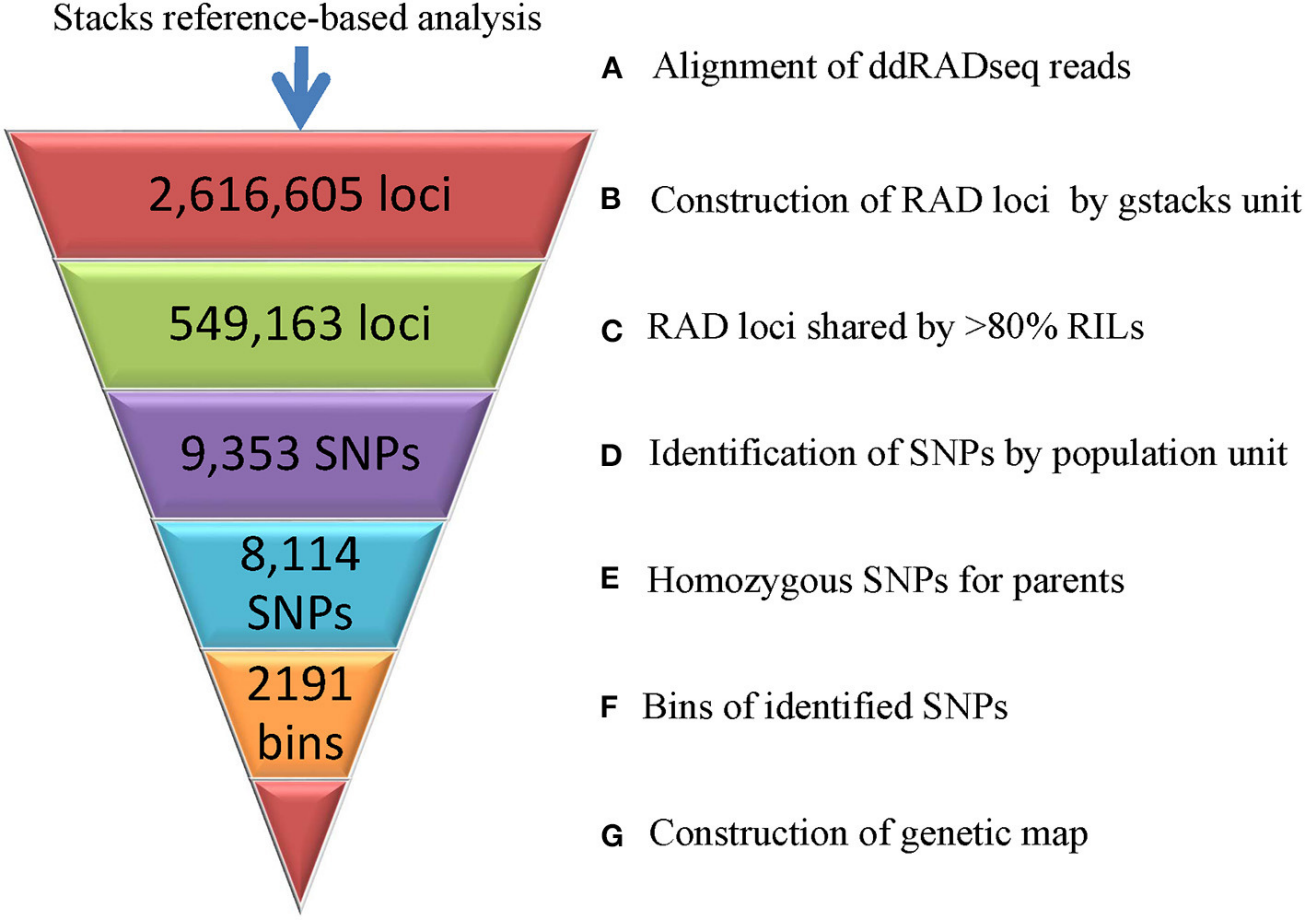
Genotyping of the RIL population based on ddRADseq data.

The 359.96 Gb ddRADseq clean data (3,712.28 M reads) of the parents and RIL population were analyzed using the recently reported genome sequences of Tifrunner as reference. A total of 2,616,605 loci were built following the pipeline of reference-based analysis of the stacks software (Figure 2). Among the RILs, the loci ranged from 492,063 to 757,190 and the sequencing depths varied from 4.17 to 13.45. After removing loci missed in more than 20% RILs, 549,163 loci were used to call SNPs. In total, 9,353 SNPs across the RIL population were obtained. A total of 8,114 SNPs showed homozygous genotypes in two parents of the RIL population. These 8,114 SNPs were clusters as 2,191 bins, among which 1,277 bins had single SNP while the other 914 bins had 2-148 adjacent SNPs (7.48 on average) with same genotypes in the RIL population.

### Construction of High-Density Genetic Map

**Figure 3:**
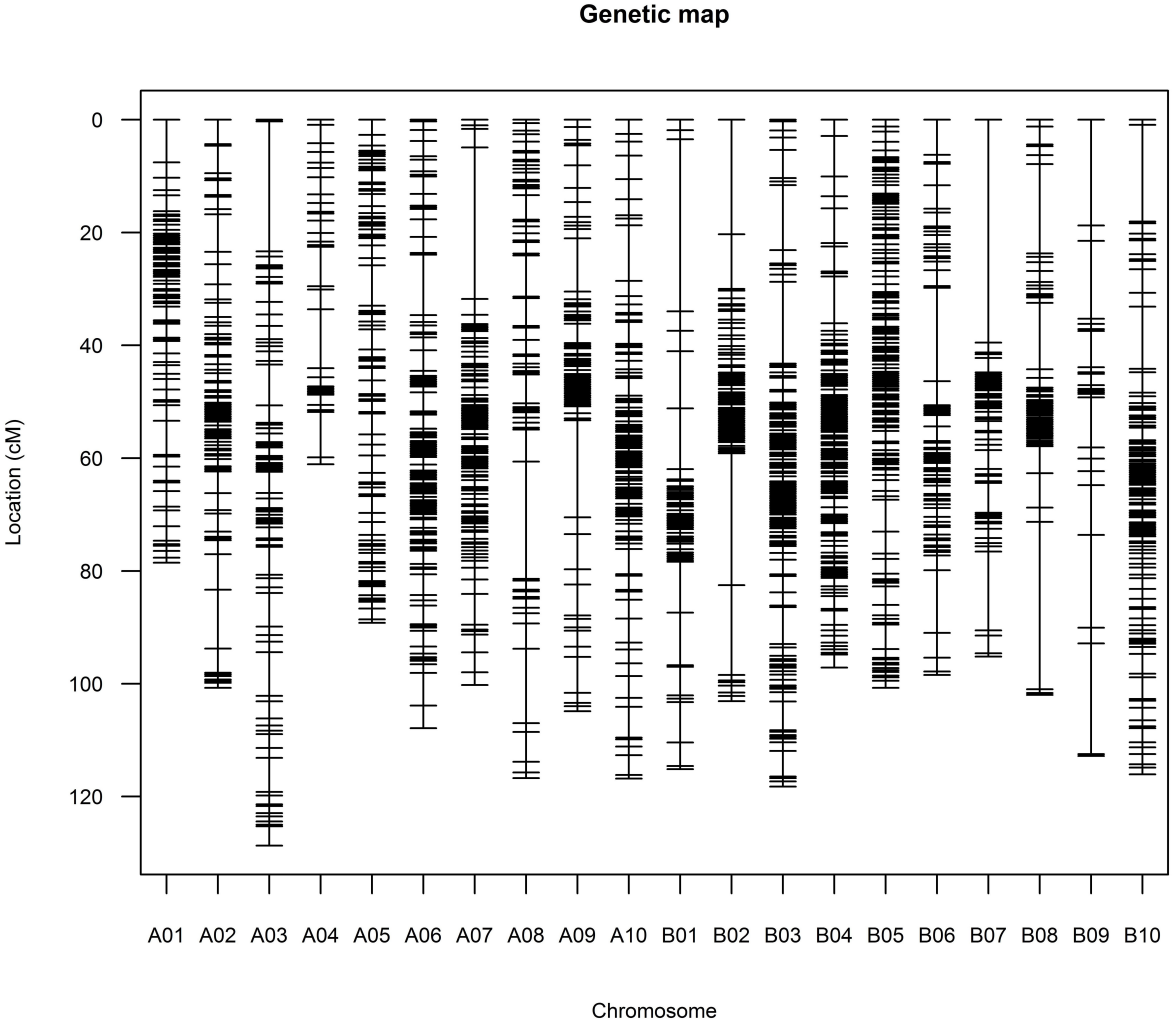
Graph presentation of the constructed high-density genetic map.

The above 2,191 bins were used to construct genetic map, and the final genetic map was consisted of 2,183 genetic loci (Figure 3, Supplementary Figure 1, Supplementary Table 2). The total length of the 20 linkage groups (LGs) was 2,063.55 cM. The 10 LGs (A01–A10) of subgenome A contained 1,050 loci with a total length of 1,004.73 cM while the 10 LGs (B01–B10) of subgenome B contained 1,133 loci spanning 1,058.82 cM (Table 2). LG B03 had the largest loci (191) and LG B09 had the least loci (25). The length of LGs ranged from 61.08 cM for A04 to 128.69 cM for A03. The average marker interval of the genetic map was 0.95 cM, ranging from 0.52 cM for B04 to 4.51 cM for B09. Half of the LGs had marker densities of <1 cM/locus, and 98.21% of the genetic distances between markers were lower than 5 cM, indicating a high degree of linkage. The largest gap between markers was 39.54 cM on LG B07, which was corresponding to a large (>60 Mb) physical gap without polymorphic SNPs between parents of the RIL population. According to the Pearson's Chi square test, segregations of 92 loci (accounting for 4.21% of the total loci) were distorted (P < 0.01, Supplementary Table 2). The 92 loci distortedly segregated to Zhonghua 6 in the A subgenome while to Xuhua 13 in the B subgenome. LG B05 contained the most of distorted loci (41), followed by A05 (29), B07 (5), A06 (3), A09 (3), B01 (3), A07 (2), B04 (2), B02 (1), B03 (1), B08 (1) and B10 (1).

**Table 2:** Description of the high-density genetic linkage map constructed in the present study.

**Linkage group**	**Number of loci**	**Length (cM)**	**Average spacing**	**Max spacing**	**SDL^**a**^**	**SDL%^**b**^**	**P1^**c**^**	**P2^**d**^**	**Reference length (Mb)**	**% of genome coverage**
A01	93	78.54	0.85	7.56	-	-	-	-	112.42	92.31
A02	115	100.71	0.88	10.44	-	-	-	-	102.98	96.52
A03	82	128.69	1.59	23.06	-	-	-	-	143.81	95.68
A04	32	61.08	1.97	10.46	-	-	-	-	128.80	88.90
A05	84	89.20	1.07	7.14	29	34.52	29	-	115.93	91.11
A06	152	107.88	0.71	10.71	3	1.97	3	-	115.50	96.57
A07	181	100.22	0.56	26.86	2	1.10	2	-	81.12	98.06
A08	66	116.74	1.80	20.78	-	-	-	-	51.90	99.00
A09	119	104.86	0.89	17.17	3	2.52	-	3	120.52	98.08
A10	126	116.82	0.93	9.85	-	-	-	-	117.09	98.05
A subgenome	1,050	1,004.73	0.96	26.86	37	3.52	34	3	1090.08	95.43
B01	79	115.15	1.48	30.53	3	3.79	-	3	149.30	99.57
B02	88	103.08	1.18	23.39	1	1.14	-	1	120.58	95.71
B03	191	118.25	0.62	14.51	1	0.52	1	-	146.73	99.42
B04	188	97.13	0.52	8.29	2	1.06	1	1	143.24	94.76
B05	176	100.73	0.58	5.65	41	23.30	-	41	160.88	95.78
B06	81	98.44	1.23	16.56	-	-	-	-	154.81	96.22
B07	55	95.19	1.76	39.54	5	9.09	-	5	134.92	99.42
B08	83	101.97	1.24	29.70	1	1.20	1	-	135.15	99.06
B09	25	112.81	4.70	19.65	-	-	-	-	158.63	98.36
B10	167	116.07	0.70	17.17	1	0.60	-	1	143.98	99.87
B subgenome	1,133	1,058.82	0.93	39.54	55	4.85	3	52	1448.21	97.82
Whole genome	2,183	2,063.55	0.95	39.54	92	4.21	37	55	2538.28	96.62

a *The number of segregation distortion loci in each linkage group (P < 0.001)*.

b *The percentage of segregation distortion loci in each linkage group (P < 0.001)*.

c *The number of loci that segregated distortedly to the male parent Zhonghua 6*.

d *The number of loci that segregated distortedly to the female parent Xuhua 13*.

### Evaluation of Genome Synteny of the High-Density Genetic Map

**Figure 4:**
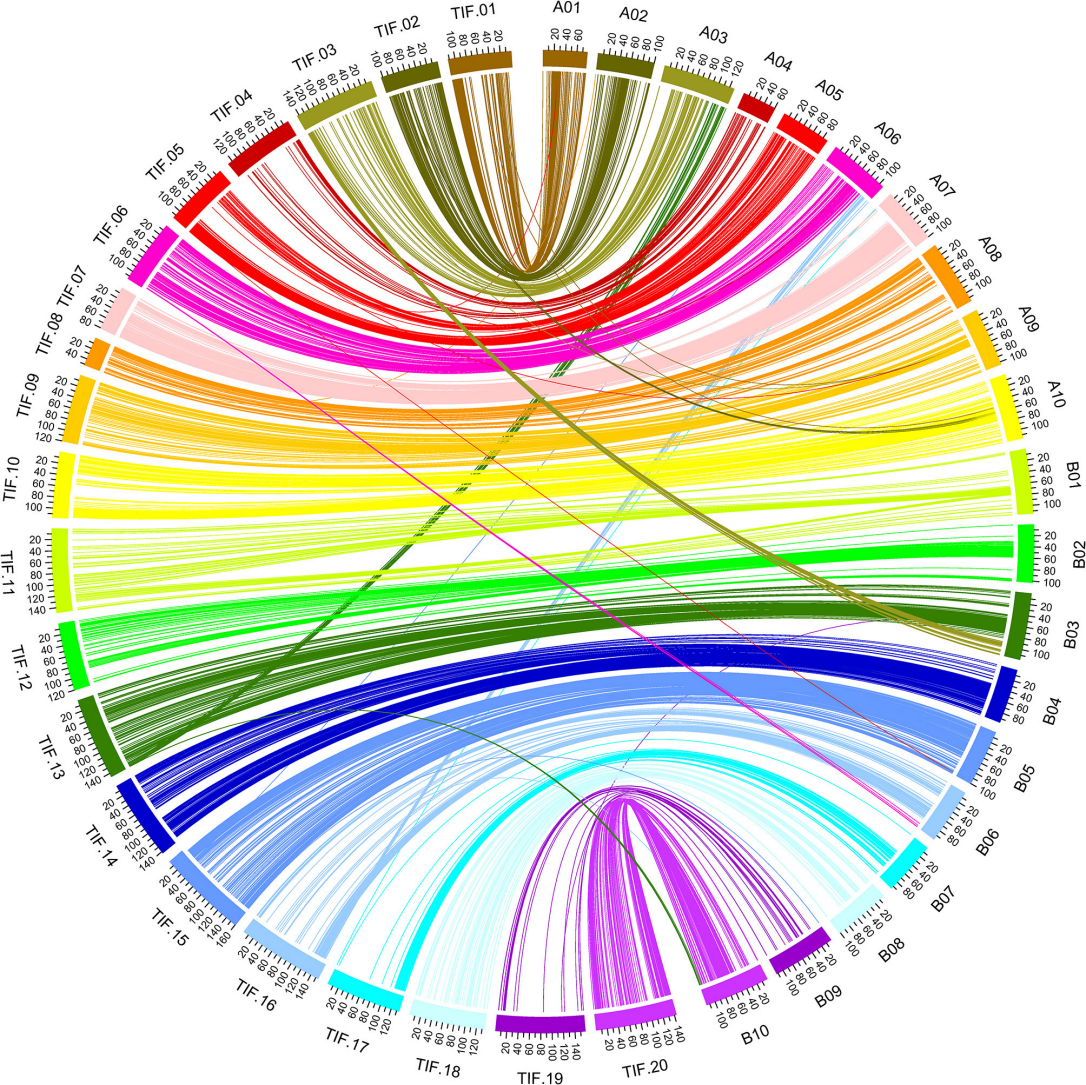
Circos graph presentation of the genome synteny between genetic map and physical map.

A total of 2,099 SNP loci (accounting for 96.15%) of the genetic map were located on same chromosomes of the reference genome of Tifrunner (Bertioli et al., 2019), and good collinearity was clearly observed for almost all chromosomes (Figure 4, Supplementary Figure 2). BLASTn analysis of the RAD-tags of the other 84 SNP loci against to genome sequences of cultivated peanut Tifrunner, Shitouqi (Zhuang et al., 2019), and Fuhuasheng (Chen et al., 2019a) as well as wild species (Bertioli et al., 2016) found that translocations occurred in different peanut varieties (Supplementary Table 3). Obviously, reciprocal translocations occurred between chromosome A03 and B03 as well as A06 and B06 in Tifrunner but not in Shitouqi, Fuhuasheng and the parents of the RIL population. Interestingly, a fragment from B10 was translocated to B03 in Tifrunner and Shitouqi but not in Fuhuasheng.

### QTL Associated With Resveratrol Content

**Figure 5:**
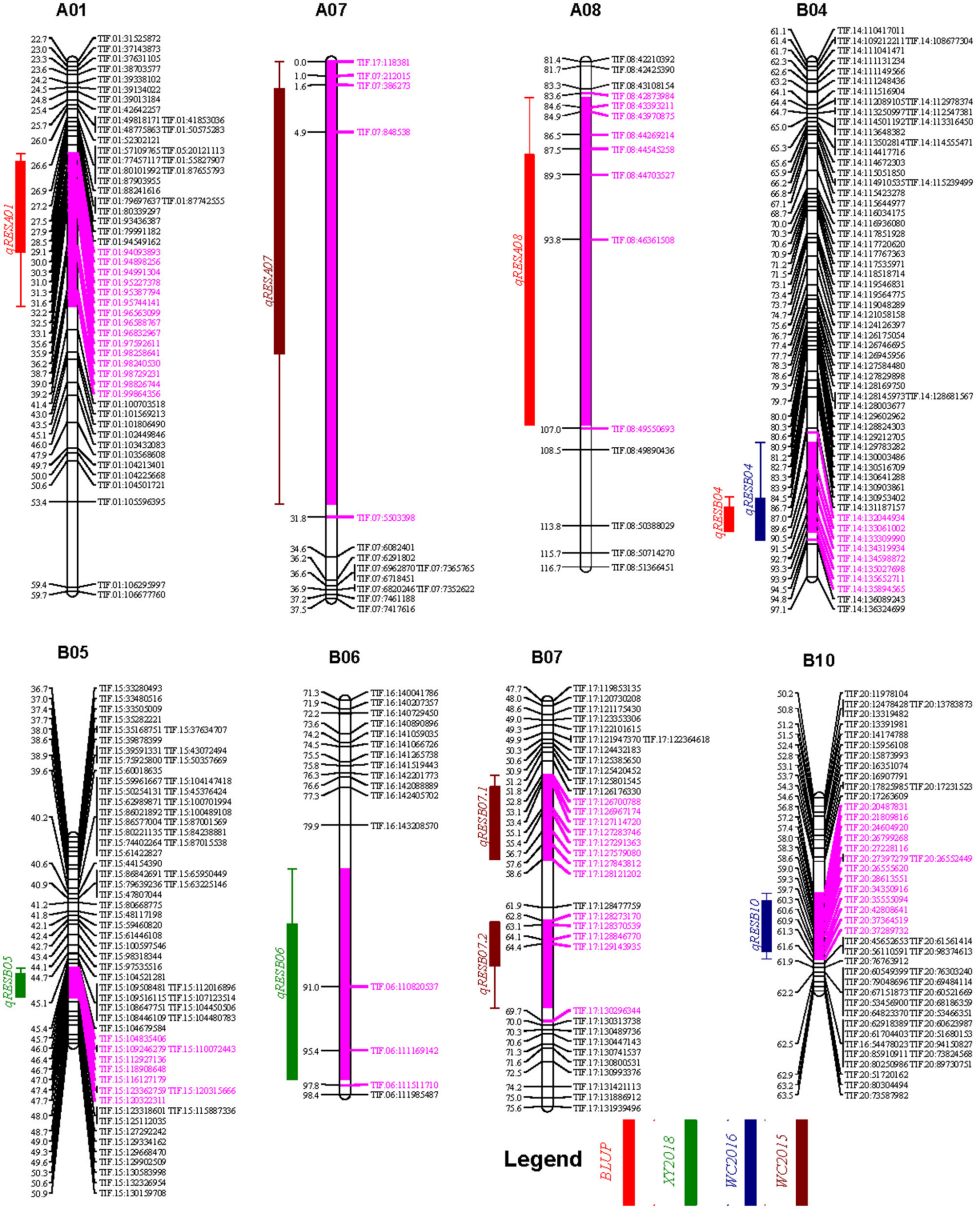
The genetic map locations of QTL identified for resveratrol contents. QTL and their 1 LOD confidential intervals (filled boxes) and 2 LOD confidential intervals (lines) were colored according to environments as shown in figure legend. The purple color was used to highlight the 2 LOD confidential intervals on chromosome bars. Bins within the 2 LOD confidential intervals were highlighted in purple color as well.

Based on the high-density genetic map, three, two, and two QTL for resveratrol content were identified the phenotyping data of the WC2016, WC2017, and XY2018 environments, respectively, while no QTL was identified in the YL2017 environment. In addition, three QTL were identified using the BLUP values across the four environments (Table 3). The *qRESB04* could be repeatly identified using the phenotyping data of the WC2016 environment as well as the BLUP values across four environments. Therefore, a total of nine QTL were identified across four environments (Table 3), and were located on eight chromosomes, namely A01, A07, A08, B04, B05, B06, B07, and B10 (Figure 5), with 5.07–8.19% phenotypic variation explained (PVE). The confidential intervals of the identified QTL varied from 2.0 cM for *qRESB05* to 30.9 cM for *qRESA07* and averaged 10.64 cM. The physical intervals of the identified QTL in the reference genomes of Tifrunner were conveniently estimated according to the names of flanking markers. The *qRESB05* and *qRESB10* were located in lower recombination regions on chromosome B05 and B10, and their physical intervals (~18.53 Mb and ~22.32 Mb, respectively) were much larger than others (0.69–6.68 Mb).

**Table 3:** Summary of QTL identified for resveratrol contents in the RIL populations across four environments.

**QTL**	**Envr ^**a**^**	**LG^**b**^**	**Position (cM)**	**LOD**	**Add^**c**^**	**PVE^**d**^**	**Genetic interval (cM)**	**Physical interval (Mb)**	**No. of genes**
*qRESA01*	BLUP	A01	32.51	2.78	2.06	5.07	10.7	5.77	433
*qRESA07*	WC2015	A07	4.91	2.54	−10.41	5.61	30.9	5.50	544
*qRESA08*	BLUP	A08	92.31	2.50	−2.08	5.32	22.9	6.68	491
*qRESB04*	WC2016	B04	93.91	2.92	11.28	6.29	6.8	3.85	216
	BLUP	B04	93.31	3.58	2.36	6.81	2.4	1.33	69
*qRESB05*	XY2018	B05	47.01	2.57	9.33	5.54	2.0	18.53	151
*qRESB06*	XY2018	B06	91.01	2.73	−9.85	6.12	14.5	0.69	43
*qRESB07.1*	WC2015	B07	55.41	3.64	12.57	8.19	5.8	1.42	63
*qRESB07.2*	WC2015	B07	63.21	2.71	10.88	6.09	5.9	2.02	96
*qRESB10*	WC2016	B10	59.41	2.76	−10.78	5.83	4.5	22.32	392

a *Environments*.

b *The constructed linkage groups*.

c *The estimated additive effects. A positive additive effect indicated male parent Zhonghua 6 as the source of alleles improving resveratrol contents, while a negative additive effect indicated that the allele for increasing resveratrol contents came from the female parent Xuhua 13*.

d *The percentage of the phenotypic variation explained by additive effect (%)*.

A total of 2,429 putative genes were retrieved within physical intervals of these QTL from the genome annotation of Tifrunner, ranging from 43 genes for *qRESB06* to 544 genes for *qRESA07* (Table 3, Supplementary Table 4). As shown in Supplementary Table 5, GO terms were retrieved for 1,410 genes from the Tifrunner genome annotation file. In terms of cellular components, cell (14.8%) was the most frequent GO term, followed by membrane (13.6%), organelle (9.6%), and protein-containing complex (6.7%) (Supplementary Figure 3). For molecular functions, binding (63.2%) was the most frequent GO term and followed by catalytic activity (47.4%) (Supplementary Figure 3). For biological processes, metabolic process (44.4%) was the most frequent GO term, followed by cellular process (35.0%), biological regulation (10.4%), and localization (9.5%) (Supplementary Figure 3). Based on the KEGG analysis, 94 enzymes that might be encoded by 161 genes were assigned to 81 KEGG pathways of various primary and secondary metabolisms (Supplementary Table 6).

## Discussion

Peanut is one of the few edible plants for human dietary intake of resveratrol. Sanders et al. (2000) reported that the resveratrol contents of 15 peanut varieties were ~196.47 μg/kg. Wang et al. (2013) detected the seeds of 102 accessions within the U.S. peanut mini-core collection and found that the resveratrol contents varied from 30 to 260 μg/kg and averaged at 100 μg/kg. In the present study, the resveratrol content in seeds of the RIL population showed similar variation range (i.e. 3.61–282.69 μg/kg). In addition, significant variations across environments were observed, including locations and years (Table 1 and Supplementary Table 1), which is consistent with the reports that varieties, geographical indications and vintage years significantly influenced the resveratrol contents in red wines (Nikfardjam et al., 2006; Pastor et al., 2019). Moreover, the broad sense heritability of resveratrol content across environments was relatively low (0.33). Therefore, attentions should be paid to not only varieties but also producing geographical locations as well as years in the production of high resveratrol peanut, and the breeders should specify their breeding programs for different target producing locations.

The availability of multiple peanut reference genomes facilitated the construction and evaluation of high-density genetic map as well as the discovery of chromosome variations. Previous studies constructed high-density genetic map through the *de-novo* approach of the stacks software (Zhou et al., 2014; Wang et al., 2018; Liu et al., 2020). However, majority of the linkage map construction software are not affordable for the large number of SNPs. The SNPs were reduced randomly or with very stringent filters (Zhou et al., 2014; Wang et al., 2018; Liu et al., 2020), which might cause the loss of useful SNPs. In the present study, using the recently reported cultivate peanut genome assembly (Bertioli et al., 2019) as reference, the previously reported ddRADseq reads (Liu et al., 2020) were re-analyzed through the reference-based pipeline of stacks. The identified SNPs were binned according to not only their genotypes in the RIL population but also physical positions on the reference genome, which effectively removed redundant SNPs and helped in the construction of high-density genetic map (Figures 2, 3). The constructed genetic map was estimated to cover 96.62% of the reference genome, and its loci number and density reached a fairly high level when compared to previous studies (Ravi et al., 2011; Qin et al., 2012; Huang et al., 2015; Chen et al., 2016, 2017, 2019b; Wang et al., 2019a). Moreover, good collinearity between the constructed genetic map with the physical map was revealed by comparing to multiple peanut reference genomes (Figure 4 and Supplementary Table 3). Notably, translocations between chromosomes, such as A03 and B03, A06 and B06, B10, and B03, were observed in present study, which was ignored in the previous study using the *de-novo* approach (Liu et al., 2020).

The resveratrol content is a complicated trait and controlled by multiple QTL from both subgenomes of peanut. In the present study, a total of nine additive QTL (5.07–8.19% PVE) for resveratrol content were identified on three chromosomes (A01, A07, and A08) of the A subgenome and five chromosomes (B04, B05, B06, B07, and B10) of the B subgenome (Table 3, Figure 5). The top five RILs had higher resveratrol contents than parents, and they possess the favored alleles of 4–7 identified QTL from both parents (Supplementary Table 7). Only one QTL, the *qRESB04*, was identified in the WC2016 environment as well as using the BLUP values across four environments, while the other nine QTL were identified only in single environment. In addition, no QTL were identified in the YL2017 environment, in which majority of RILs skewed obviously toward low resverartrol contents (Figure 1). These phenomena were consistent with the fact that environments and QTL × environment interactions had significant influences in peanut resveratrol content. A pool of 2,429 putative genes were retrieved from the corresponding physical intervals of these QTL in the Tifrunner genome (Bertioli et al., 2019), and many of them involved in diverse primary and secondary metabolic pathways. However, target genes influencing resveratrol content could not be predicted in the present study. Further studies, e.g., transcriptome/metabolome analysis, might provide more evidence to identify key metabolites and candidate genes associated with resveratrol synthesis in peanut. Moreover, the peanut synthase of resveratrol (STS) (Shomura et al., 2005) might be located at ~13 Mb on chromosome B04 or ~11 Mb on A04, which were outside of the identified QTL. Therefore, resveratrol, as a secondary metabolite, might be synthesized by STS under the influence of various upstream biological processes, and it would be a challenge to breed high resveratrol peanut varieties.

In conclusion, the present study constructed a high quality genetic map and identified nine QTL with main effects for resveratrol content using a RIL population across four environments. These QTL lay a foundation for the future marker assisted selection (MAS) in the improvement of resveratrol content. In future studies, a wide range screening of germplasms in multiple producing environments should be conducted to determine the range of resveratrol contents in peanut seeds and identified elite accessions for genetic studies and breeding utilization. In addition, more studies could be conducted to evaluate the resveratrol contents, variations as well as their relationships in different peanut tissues, which might help developing diverse high resveratrol peanut products. Along with the reducing cost of high-throughput sequencing or genotyping, genome-based trait prediction models (Pandey et al., 2020) for the resveratrol content could be build and might be used in genomic selection (Crossa et al., 2017) to breed high resveratrol varieties.

## Data Availability Statement

The original contributions presented in the study are included in the article/Supplementary Material, further inquiries can be directed to the corresponding author/s.

## Author Contributions

HL, YL, BL, and HJ conceived, designed, and supervised the experiments. XR, LY, DH, YL, BL, and HJ developed the RIL population. JG, BY, WC, HZ, XZ, YC, NL, and LH conducted field trials and phenotyping of resveratrol contents by HPLC. HL performed the construction of high-density genetic map from sequencing data and interpreted the results. HL prepared the first draft and YL, BL, and HJ contributed to the final editing of manuscript. All authors read and approved the final manuscript.

## Conflict of Interest

The authors declare that the research was conducted in the absence of any commercial or financial relationships that could be construed as a potential conflict of interest. The reviewer MY declared a past co-authorship with several of the authors BL and HJ to the handling editor.
