# Supplementary figures and images for: Construction of ddRADseq-Based High-Density Genetic Map and Identification of Quantitative Trait Loci for Trans-resveratrol Content in Peanut Seeds
Source: Front Plant Sci. 2021 Mar 18;12:644402. doi: 10.3389/fpls.2021.644402 (PMC8044979; doi:10.3389/fpls.2021.644402)

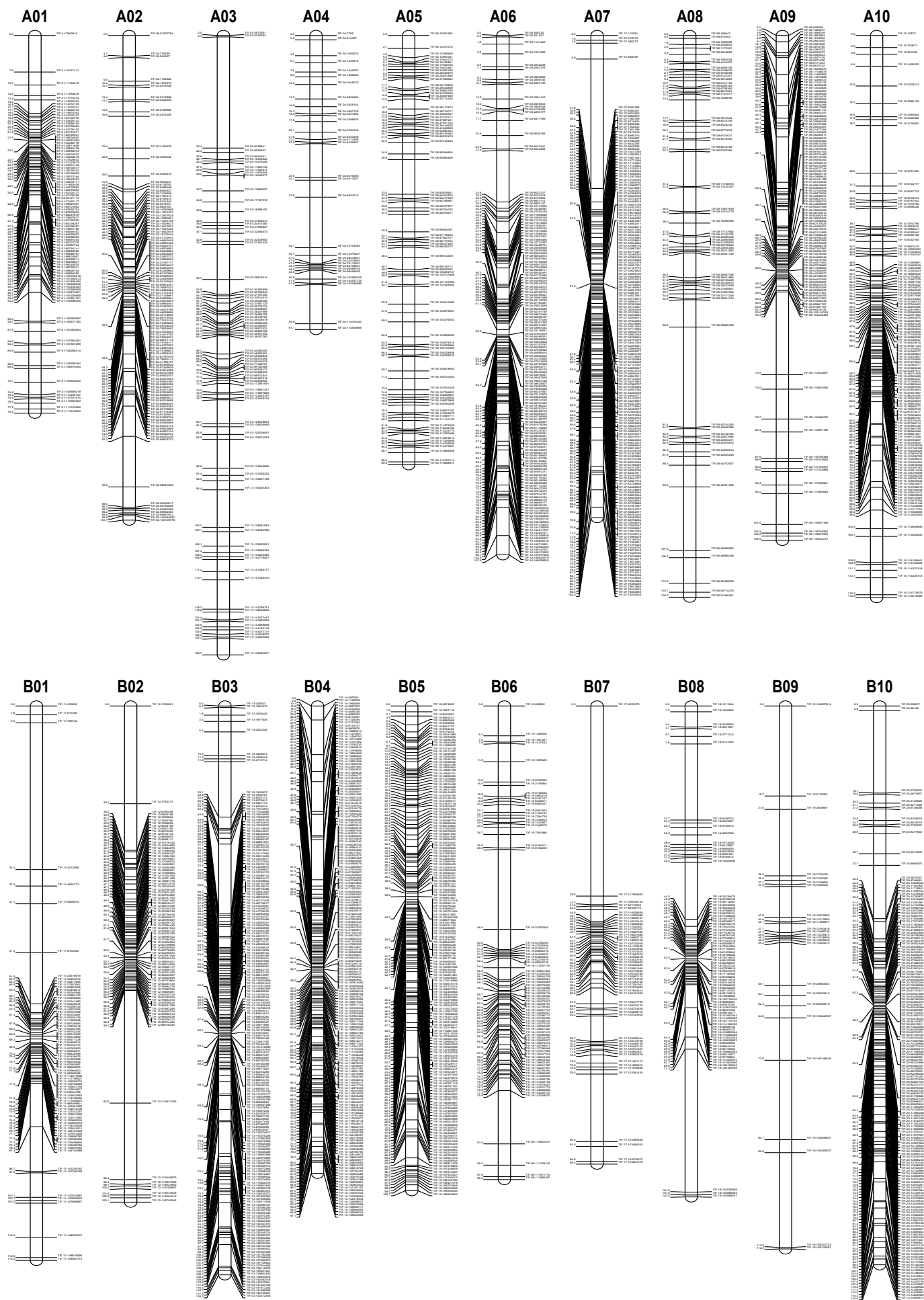

**Supplemental Figure 1.** Graph presentation of the constructed genetic map with SNP names.

Supplement: Supplementary file 8 [file Image_1.PDF]

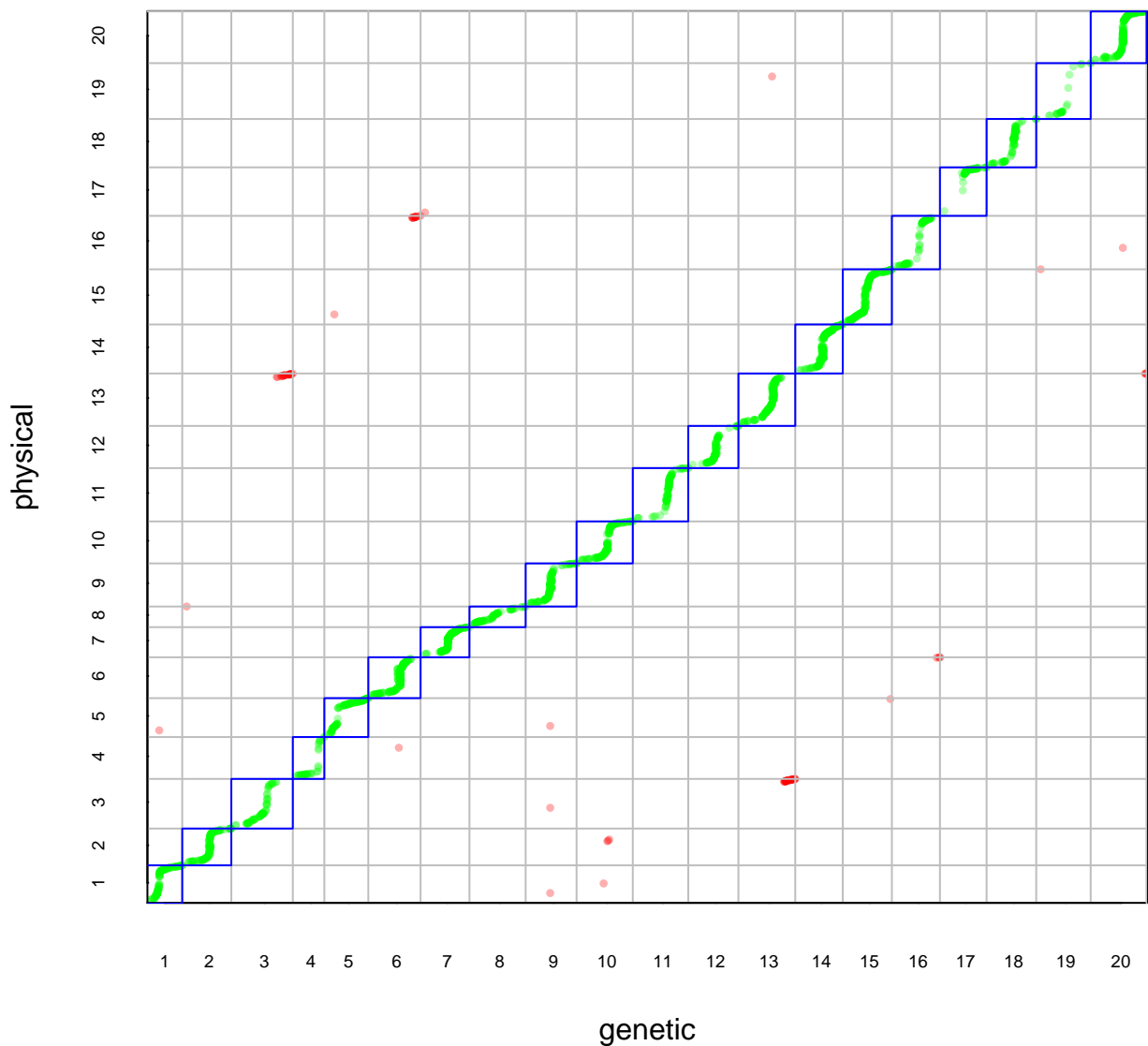

**Supplemental Figure 2.** Dotplot of the genome synteny between genetic map and physical map.

Supplement: Supplementary file 9 [file Image_2.PDF]
