# Supplementary material for: Construction of ddRADseq-Based High-Density Genetic Map and Identification of Quantitative Trait Loci for Trans-resveratrol Content in Peanut Seeds
Source: Front Plant Sci. 2021 Mar 18;12:644402. doi: 10.3389/fpls.2021.644402 (PMC8044979; doi:10.3389/fpls.2021.644402)
Supplement: Supplementary file 10 [file Image_3.PDF]

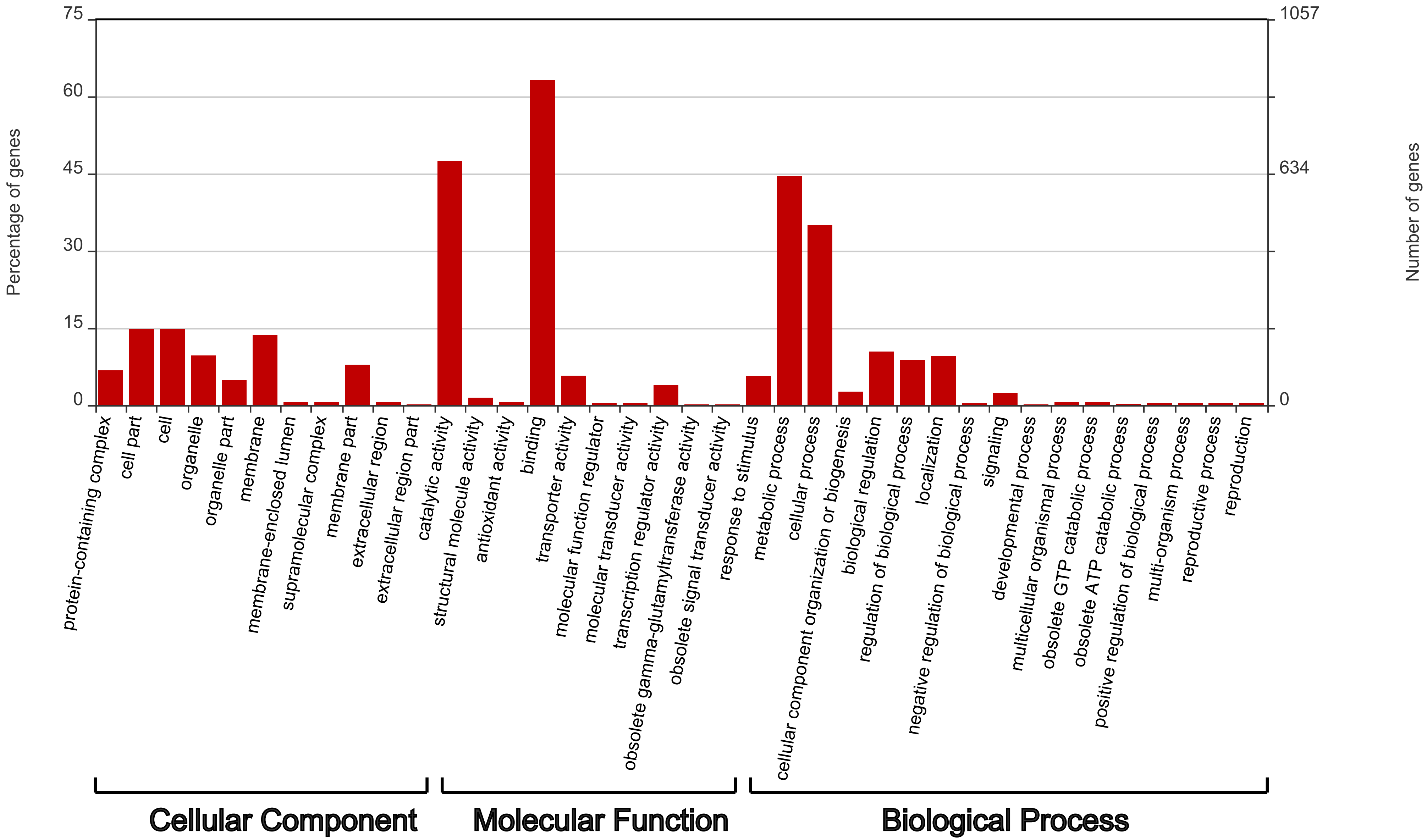

Supplemental Figure 3. WEGO plot of the GO terms for putative genes within confidential intervals of QTLs for resveratrol contents
